# Venomic Analysis of the Poorly Studied Desert Coral Snake, *Micrurus tschudii tschudii*, Supports the 3FTx/PLA_2_ Dichotomy across *Micrurus* Venoms

**DOI:** 10.3390/toxins8060178

**Published:** 2016-06-07

**Authors:** Libia Sanz, Davinia Pla, Alicia Pérez, Yania Rodríguez, Alfonso Zavaleta, Maria Salas, Bruno Lomonte, Juan J. Calvete

**Affiliations:** 1 Laboratorio de Venómica Estructural y Funcional, Instituto de Biomedicina de Valencia, CSIC, Jaime Roig 11, Valencia 46010, Spain; 2 Departamento Academico de Ciencias Celulares y Moleculares, Facultad de Ciencias y Filosofía, Universidad Peruana Cayetano Heredia, Lima 31, Peru; 3 Instituto Nacional de Salud, Ministerio de Salud, Lima 11, Peru; 4 Instituto Clodomiro Picado, Facultad de Microbiología, Universidad de Costa Rica, San Jose 11501, Costa Rica

**Keywords:** *Micrurus tschudii tschudii* venom, venomics, snake venom proteome, three-finger toxin, snake venom phospholipase A_2_, mass spectrometry

## Abstract

The venom proteome of the poorly studied desert coral snake *Micrurus tschudii tschudii* was unveiled using a venomic approach, which identified ≥38 proteins belonging to only four snake venom protein families. The three-finger toxins (3FTxs) constitute, both in number of isoforms (~30) and total abundance (93.6% of the venom proteome), the major protein family of the desert coral snake venom. Phospholipases A_2_ (PLA_2_s; seven isoforms, 4.1% of the venom proteome), 1–3 Kunitz-type proteins (1.6%), and 1–2 l-amino acid oxidases (LAO, 0.7%) complete the toxin arsenal of *M. t. tschudii*. Our results add to the growing evidence that the occurrence of two divergent venom phenotypes, *i.e.*, 3FTx- and PLA_2_-predominant venom proteomes, may constitute a general trend across the cladogenesis of *Micrurus*. The occurrence of a similar pattern of venom phenotypic variability among true sea snake (Hydrophiinae) venoms suggests that the 3FTx/PLA_2_ dichotomy may be widely distributed among Elapidae venoms.

## 1. Introduction

New World genus *Micrurus* (Elapidae) (Wagler, 1824) [1] represents a monophyletic clade of some 80 currently recognized species of venomous coral snakes [2,3,4,5,6,7,8], although the topology of the tree is still unresolved. Coral snakes are widely distributed in tropical and subtropical regions from the southern United States to northeastern Argentina, including several continental islands inhabited by endemic forms [2,3]. Coral snakes are considered by herpetologists to be among the most beautiful snakes of the planet, as they are adorned with unique combinations of red-, black-, and yellow-colored banding. Based on their body ring color pattern characteristics, tail proportion with respect to body length, and hemipenial morphology, coral snakes are traditionally arranged in four species groups: a tricolored monadal group, a bicolored group, a Central American tricolored triadal group, and a South American triadal group [3,9,10,11,12]. The monadal group comprises long-tailed species with a single black ring separating the white and red rings; the bicolored group contains short-tailed species with white or red rings separated by black rings; the Central American triadal group taxa are long-tailed species with three black rings separated by white rings between red rings; the South American triadal group is represented by short-tailed species with the same color pattern as the Central American triadal group species but is restricted geographically to South America, extending from Panama to southern Argentina. 

In the last decade, the increasing application of omics techniques to the study of snake venoms has greatly enhanced our knowledge on their composition, evolution, biological activities, and clinical effects [13,14,15,16,17]. However, the venoms of only a handful of the vast number (~130) of species and subspecies that constitute the genus *Micrurus* have been the subject of proteomic studies (consult Table 2 of [18]). To understand this fact it should be taken into consideration that, despite producing among the most potent neurotoxic venoms of any New World snake, bites and fatalities by coral snakes are very rare. This is facilitated by the fact that coral snakes are not aggressive; when confronted by humans, coral snakes will almost always attempt to flee, and bite only as a last resort. On the other hand, coral snakes generally inhabit sparsely populated areas and are thus infrequently encountered, and they have short fangs that cannot penetrate thick leather clothing. Consequently, envenomings by coral snakes are less frequent than those produced by sympatric pitvipers, by far the most dangerous snakes of South America, accounting for less than 3% of the snakebites recorded in the Americas [19,20,21]. 

**Figure 1:**
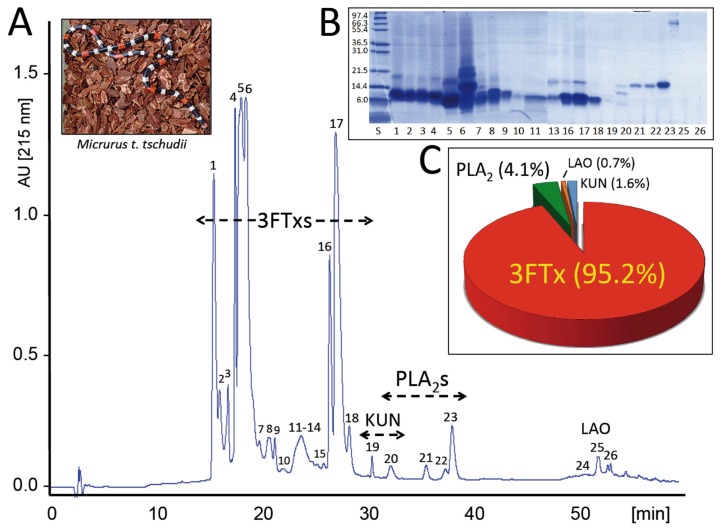
Panel (**A**) reverse-phase HPLC separation of the venom proteins from *M. t. tschudii*. Photo credit: Dr. Med. Vet. Gualberto Marcas Cáceres, Centro Nacional de Productos Biológicos, Instituto Nacional de Salud, Ministerio de Salud, Perú; Panel (**B**) SDS-PAGE of the isolated chromatographic fractions run under reduced conditions; Panel (**C**) displays the relative abundance (in % of the total venom proteins) of the toxin families found in *M. t. tschudii* venom.

The desert coral snake *M. tschudii tschudii* (January 1858) [22], one of the lesser-studied species of the genus *Micrurus*, belongs to the South American triad coral snakes [11,12,23]. Named after the Swiss naturalist, explorer and diplomat Johann Jakob von Tschudi (1818–1889), who traveled extensively in South America and published important works in herpetology, *M. t. tschudii* is a small (adults average 45 to 55 cm in length), tri-colored (Figure 1), mainly diurnal and terrestrial coral snake, found in tropical deciduous forest, dry tropical forest, and thorn scrub, mainly along watercourses, from near sea level to 1450 m elevation, in the western slopes of the Andes on the semi-arid Pacific coast of South America, from southern Ecuador to southwestern Peru [2,3,24]. The desert coral snake feeds on geckkonid lizards (*Phyllodactilus* spp.), Amphisbaenids (*A. occidentalis*), and colubrids of the species *Mastigodryas haathi* [3]. However, available literature about its reproduction and activity is scarce, and in particular virtually nothing (not even a single entry in PubMed) is known about its venom. Here, we have applied a venomics approach [13,14] to gain an insight into the spectrum of toxins that make up the venom proteome of *M. t. tschudii*.

## 2. Results and Discussion

### 2.1. The Venom Proteome of the Desert Coral Snake

The venom of *M. t. tschudii* was fractionated into 26 RP-HPLC fractions (Figure 1A). Each chromatographic fraction was analyzed by SDS-PAGE (Figure 1B), and the protein bands were excised and submitted to mass spectrometric analysis. The MS/MS data, listed in Supplementary Table S1, resulted in the identification of ≥38 proteins belonging to only four snake venom protein families: three-finger toxin (3FTx), phospholipase A_2_ (PLA_2_), Kunitz-type, and l-amino acid oxidase (LAO) (Table 1). The relative abundances of *M. t. tschudii* venom proteins and toxin families are displayed, respectively, in Table S1 and Figure 1C. The 3FTxs constitute, by far, both in number of isoforms (~30) and total abundance (93.6% of the total venom proteins), the major protein family of the desert coral snake venom. Seven PLA_2_ isoforms, 1–3 Kunitz-type proteins, and 1–2 LAO molecules (accounting, respectively, for 4.1%, 1.6%, and 0.7% of the venom proteome) complete the toxin arsenal of the desert coral snake.

The 3FTx and PLA_2_ molecules are hallmark components of the venoms of Elapidae. Catalytically active PLA_2_ molecules typically exhibit presynaptic neurotoxic activity, myotoxic activity, or both, although some forms exhibit antiplatelet activity [25,26]. Despite their pronounced structural similarity, members of the 3FTx family exhibit a wide variety of pharmacological effects including postsynaptic neurotoxicity, cytotoxicity, cardiotoxicity, and anticoagulant, and antiplatelet activities [27,28]. In addition, l-type Ca^2+^ channel antagonists of the 3FTx family may act synergistically with muscarinic three-finger toxins to promote hypotension [29]. Kunitz-type serine protease inhibitor isolated from elapid venoms blocked the activity of a range of serine proteases, producing an antihemorrhagic effect [30]. Non-covalent PLA_2_-KUN complexes have been characterized from venom of some *Micrurus* species [18 and references cited]. These complexes, originally discovered in the venom of the Texas coral snake *M. tener*, are target acid-sensing receptors ASIC1a/2 evoking pain [31,32]. There are many examples of venom-derived toxins that elicit notoriously intense pain [33]. Pain response may cause paralysis and serve as a warning signal for discouraging potentially threatening predators by triggering lasting acute physiological distress. The l-amino acid oxidases are flavoenzymes that catalyze oxidative deamination of l-amino acids to form corresponding α-keto acids, hydrogen peroxide and ammonia. LAOs are thought to contribute to the toxicity of the venom due to the production of hydrogen peroxide during the oxidation reaction [34,35], although their contribution to the envenoming process remains elusive.

The low yield of venom and the challenge of long-term maintenance of the desert coral snake in captivity precluded a detailed toxicovenomics analysis of the venom components of this poorly studied *Micrurus* species.

### 2.2. 3FTx- and PLA_*2*_-Predominant Venom Proteomes among Coral Snakes

Despite their extremely adaptive ecological radiation, coral and sea snakes show virtually identical rates of body size evolution to other elapids, suggesting that their diversification may be correlated with phenotypic traits other than body size [36]. Given the central role that diet has played in the adaptive radiation of snakes [37], venom represents a key adaptation that has played an important role in the diversification of these animals [38,39]. Growing evidence, including the venomic analysis of the desert coral snake here reported, supports the view that the occurrence of two divergent venom phenotypes, *i.e.*, 3FTx- and PLA_2_-predominant venom proteomes, may constitute a general trend across *Micrurus* cladogenesis [18,40]. Thus, *M. alleni* (77% 3FTx *vs.* 11% PLA_2_) and *M. mosquitensis* (56% PLA_2_, 22% 3FTx) [18]; *M. corallinus* (82% 3FTx, 12% PLA_2_) [41]; *M. nigrocinctus* (38% 3FTx, 48% PLA_2_) [42]; *M. fulvius* (21% 3FTx, 65% PLA_2_) [43,44]; *M. tener* (38% 3FTx, 46% PLA_2_) [45]; *M. dumerilii* (28% 3FTx, 52% PLA_2_) [40]; and *M. clarki* (48.2% 3FTx, 36.5% PLA2) [46] all are species included in the well-supported monophyletic monadal clade [3,10,47,48]. The 22 valid species of coral snakes classified in the South American triadal group, to which *M. t. tschudii* belongs, also form a strongly supported clade [3,11,12,23]. Biochemical and/or proteomic analyses have been reported for the venoms of *M. altirostris* (80% 3FTx, 14% PLA_2_) [44], *M. surinamensis* (3FTx-predominant venom) [49], *M. frontalis* (3FTx-predominant venom) [50], *M. pyrrhocryptus* (3FTx-rich venom proteome) [51], and *M. clarki* (moderately 3FTx-predominant venom) [46]. On the other hand, the venom proteomes of *M. multifasciatus* and *M. mipartitus* from the bicolor species group are both dominated by 3FTxs [52]. 

Experiments in mice determined the Median Lethal Dose (LD_50_) for *M. t. tschudii* venom at 10.5 µg/mouse (7.4–13.8 µg/mouse, 95% confidence limits) by the i.p. route. This figure corresponds to 0.62 µg venom/g of mouse body weight (0.44–0.81 µg/g, 95% confidence limits) (Table 1). Comparison of reported median lethal doses (LD_50_) clearly shows that 3FTx-rich and PLA_2_-rich *Micrurus* venoms are not statistically different in terms of lethality to mice (Table 1). Although a more meaningful correlation would require comparing LD_50_ values for their natural prey, current data suggest that *Micrurus* venoms may have evolved under the action of balancing selection [56]. 

**Figure 2:**
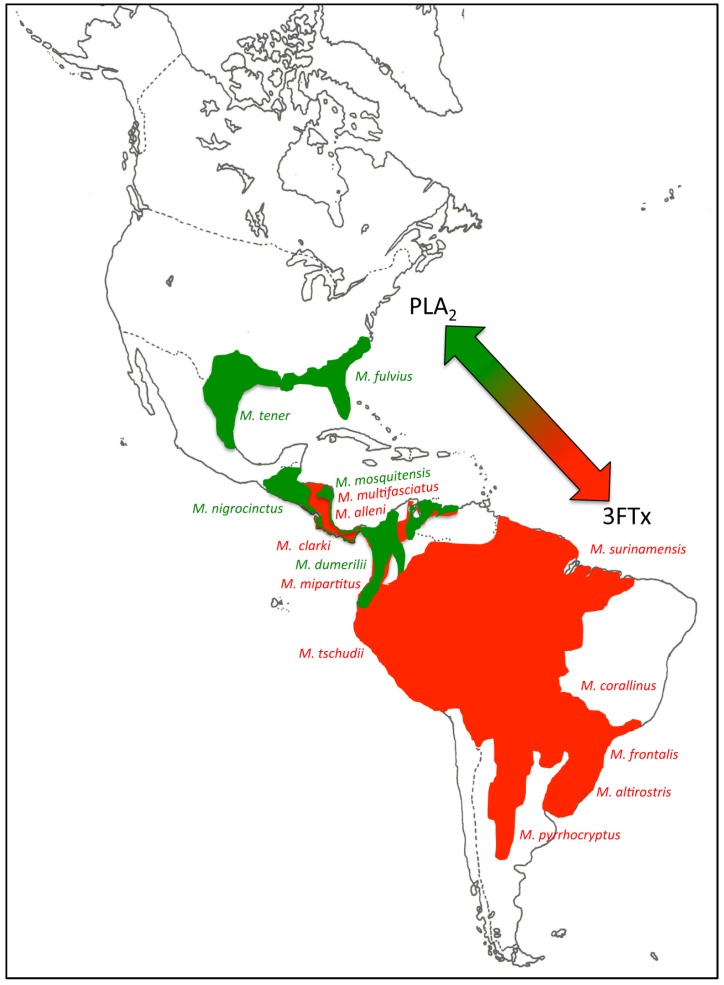
Geographic distribution of *Micrurus* species for which quantitative estimation of proteome compositions have been reported in the literature (Table 1). Distribution ranges were adapted from [3] and The Reptile Database (http://www.reptile-database.org), and are color-coded: green, PLA_2_-rich venom phenotype; red, 3FTx-predominant venom composition. The arrow highlights the trend towards diverging venom phenotypes along the *Micrurus* north-south dispersal, suggesting the epicenter of the divergence in Mesoamerica.

Margres and coworkers [44] have detected strong evidence of positive selection for the 3FTx and PLA_2_ toxin families of *M. fulvius*. This accelerated evolution is most likely due to their direct involvement in fitness. Figure 2 shows a clear geographical distribution of PLA_2_- and 3FTx-predominant *Micrurus* venoms along their north-south dispersal axis, further supporting the adaptive nature of this phenotypic dichotomy. 

### 2.3. Tracing the Evolutionary Origin of the 3FTx/PLA_*2*_ Dichotomy across Elapidae

Elapid snakes are a relatively young group. The crown elapid radiation is approximately 38 million years (My) old, yet elapid snakes exhibit some of the highest diversification rates in reptiles [57]. In particular, two clades, *Hydrophis* and *Micrurus*, show anomalously high rates of diversification within Elapidae [36]. The Asian coral snake genus *Calliophis* emerges as monophyletic and the sister group to all other American and Asian coral snakes [47], suggesting an Asiatic origin for the common ancestor of these elapids [58]. Coral snakes diversified around 30–25 My ago, and sea snakes (Hydrophiini) are approximately 16 My old [36]. Estimated divergence times suggest that Hydrophiini is a young and rapidly speciating clade that had a common ancestor ~8 My ago, although the majority of extant lineages diversified more recently, over the last ~1.5–3.5 My [36,59].

A similar pattern of venom phenotypic variability has been documented by the proteomic analysis of true sea snake (Hydrophiinae) venoms. Hence, the venom of *A. laevis* comprises mainly PLA_2_ (71%) and 3FTxs (25%) [60], whereas the venoms of *H. schistosus* [61], *H. cyanocinctus* [62], and *P. platura* [63] all share 3FTx-rich phenotypes (*i.e.*, 70.5% 3FTx/26.5% PLA_2_; 81% 3FTx/19% PLA_2_; and 50% 3FTx/33% PLA_2_, respectively). Transcripts for 3FTx and PLA_2_ molecules also represent the two main constituents of the venom gland toxin-encoding transcriptomes of *Acalyptophis peronii* (77.5% 3FTx/17.5 PLA_2_) and *Lapemis curtus* (92.4% 3FTx; 7.5% PLA_2_) [64].

The evolutionary origin and adaptive relevance of the puzzling 3FTx/PLA_2_ dichotomy remains elusive since 3FTx- and PLA_2_-predominant venoms are scattered through the phylogenetic tree of *Micrurus*. The dominant protein families in the venom proteome of *C. bivirgata flavicep*s are PLA_2_ (41%), 3FTx (22.6%) and SVMP (18.7%) [65], suggesting that the ancestor venom phenotype of coral snakes might have been of the PLA_2_-predominant type. Alternatively, 3FTx- and PLA_2_-rich venom proteomes may represent pedomorphic and ontogenetic traits, as has been documented in *Crotalus*. Rattlesnake venoms belong to one of two distinct phenotypes, which broadly correspond to type I (high levels of SVMPs and low toxicity, LD_50_ > 1 mg/g mouse body weight) and type II (low metalloproteinase activity and high toxicity, LD_50_ < 1 mg/g mouse body weight) [66]. In Neotropical rattlesnakes, the adaptive pressure for type I to type II transition was the gain of neurotoxicity and lethality to rodents, and this transition represents a miRNA-modulated pedomorphic trait that correlates with the increased concentration of crotoxin along the axis of *Crotalus* radiation in South America [67,68]. In Nearctic species, such as *C. s. scutulatus* (Css), the venom phenotype changes geographically from SVMP-rich to Mojave toxin-rich (type-II) as one moves from south central to southeastern Arizona, with a transitional zone between the SVMP and Mojave toxin phenotypes [69]. Understanding the phylogenetic origin of the 3FTx-rich and PLA_2_-predominant venom phenotypes across *Micrurus*, and whether there is parallelism between the 3FTx/PLA_2_ (*Micrurus*) and the type I/type II (*Crotalus*) venom dichotomies, requires genus-wide profiling of the venom proteomes and the venom gland transcriptomes of adult and juvenile *Crotalus* and *Micrurus* congeneric specimens. 

## 3. Concluding Remarks and Perspectives

While genomic studies on model organisms have been widely applied to identify traits involved in maintaining functional genetic variation, few studies have drawn the links between genotype, phenotype, and fitness, and the environmental pressures that act to maintain variation that affects organismal phenotypes [70,71]. Venom represents a key adaptive trophic trait that has played an important role in the radiation of advanced snakes, and thus could be a particularly powerful model system to investigate players and mechanisms of adaptive variation at the phenotype level [17,70,72]. In this regard, structural and functional studies of *Micrurus* venoms point to balancing selection as the mechanism acting to maintain a 3FTx/PLA_2_ venom dichotomy. The realization that this 3FTx/PLA_2_ dichotomy may have deep roots in the evolution of coral snakes may also have important translational implications. Thus, both types of venoms should be part of immunization mixtures aimed at generating broad-spectrum antivenoms. 

## 4. Materials and Methods 

### 4.1. Venom

Adult *M. t. tschudii* specimens were caught in the Peruvian Pacific coast regions of La Libertad, Ancash, and Lima, and kept in captivity at the serpentarium of the Instituto Nacional de Salud, Lima, Perú. Venom from nine adult *M. t. tschudii* specimens was collected and pooled during the first three months of captivity. Venom was lyophilized and stored at −20 °C until used. 

### 4.2. Isolation and Characterization of Venom Proteins

First 0.5 milligrams of crude, lyophilized venom were dissolved in 200 μL of 5% acetonitrile in water containing 0.1% trifluoroacetic acid (TFA), centrifuged to remove debris, and separated by reverse-phase HPLC using a Teknokroma Europa Protein 300 C18 (0.4 cm × 25 cm, 5 μm particle size, 300 Å pore size) column and an LC 1100 High Pressure Gradient System (Agilent Technologies, Santa Clara, CA, USA) equipped with DAD detector and micro-Auto-sampler [73]. The flow rate was set to 1 mL/min and the column was developed with a linear gradient of 0.1% TFA in water (solution A) and acetonitrile (solution B) using the following column elution conditions: isocratically (5% B) for 5 min, followed by 5%–25% B for 10 min, 25%–45% B for 60 min, and 45%–70% for 10 min. Protein detection was carried out at 215 nm with a reference wavelength of 400 nm. Fractions were collected manually, dried in a vacuum centrifuge (Savant), and redissolved in water, and submitted to molecular mass determination using a SYNAPT^®^ G2 High Definition Mass Spectrometry System (Waters Corp., Milford, MA, USA), and SDS-PAGE analysis in 15% polyacrylamide gels, under reducing and non-reducing conditions. Gels were stained with Coomassie Brilliant Blue R-250 (Sigma-Aldrich, St. Louis, MO, USA).

### 4.3. Characterization of the Venom Peptidome and Proteome

Electrophoretic protein bands were excised from a Coomassie Brilliant Blue-stained SDS-PAGE gel and subjected to in-gel reduction (10 mM dithiothreitol) and alkylation (50 mM iodoacetamide), followed by overnight sequencing-grade trypsin digestion (66 ng/μL in 25 mM ammonium bicarbonate, 10% acetonitrile; 0.25 μg/sample) in an automated processor (ProGest Protein Digestion Workstation, Genomic Solution Ltd., Cambridgeshire, UK) following the manufacturer’s instructions. Tryptic digests were dried in a SpeedVac (Savant™, Thermo Scientific Inc., West Palm Beach, FL, USA), redissolved in 15 μL of 0.1% formic acid in water, and submitted to LC-MS/MS. To this end, tryptic peptides were separated by nano-Acquity UltraPerformance LC^®^ (UPLC^®^, Waters Corporation, Milford, MA, USA) using BEH130 C18 (100 μm × 100 mm, 1.7 μm particle size) column in-line with a SYNAPT^®^ G2 High Definition Mass Spectrometry System (Waters Corp., Milford, MA, USA). The flow rate was set to 0.6 μL/min and the column was developed with a linear gradient of 0.1% formic acid in water (solution A) and 0.1% formic acid in acetonitrile (solution B), isocratically 1% B for 1 min, followed by 1%–12% B for 1 min, 12%–40% B for 15 min, 40%–85% B for 2 min. Doubly and triply charged ions were selected for collision-induced dissociation (CID) MS/MS. Fragmentation spectra were interpreted (a) manually (*de novo* sequencing); (b) using the on-line form of the MASCOT program at http://www.matrixscience.com against NCBInr database, a comprehensive, non-identical protein database compiled from GenBank CDS translations, PIR, SwissProt, PRF, and PDB; and (c) processed in Waters Corporation’s (Milford, MA, USA) ProteinLynx Global SERVER 2013 version 2.5.2. (with Expression version 2.0) and the generated .pkl peak list files were exported to MASCOT for protein identification against the NCBInr database. MS/MS mass tolerance was set to ±0.6 Da. Carbamidomethyl cysteine and oxidation of methionine were selected as fixed and variable modifications, respectively. Cut-off for MASCOT reporting was set to top 10 hits All MASCOT identifications were manually verified. Amino acid sequence similarity searches were performed against the NCBInr and UniProtKB databases using the BLASTP program implemented in the WU-BLAST2 search engine at http://www.bork.embl-heidelberg.de.

The relative abundances (expressed as percentage of the total venom proteins) of the different protein families were calculated as the ratio of the sum of the areas of the reverse-phase chromatographic peaks containing proteins from the same family to the total area of venom protein peaks in the reverse-phase chromatogram [14,74]. When more than one protein band was present in a reverse-phase fraction, their proportions were estimated by densitometry of Coomassie-stained SDS-polyacrylamide gels using ImageJ version 1.47 (Free Software Foundation, Boston, MA, USA) (http://rsbweb.nih.gov/ij). Conversely, the relative abundances of different proteins contained in the same SDS-PAGE band were estimated based on the relative ion intensities of the three more abundant peptide ions associated with each protein by MS/MS analysis. Finally, protein family abundances were estimated as the percentages of the total venom proteome.

### 4.4. Determination of LD_50_ for Mice

Animal experiments were performed in accordance with protocols approved by the Institutional Committee for the Care and Use of Laboratory Animals of the University of Costa Rica (CICUA 041-15, 21 October 2015), using CD-1 mice of either sex, provided by Instituto Clodomiro Picado. To evaluate the lethal activity, various doses of *M. t. tschudii* venom, dissolved in 200 μL of PBS, were injected into groups of five CD-1 mice (16–18 g body weight), by the intraperitoneal (i.p.) route. Deaths were scored over a 48 h period and the median lethal dose (LD_50_) was calculated by probits [75].

## Figures and Tables

**Table 1:** Comparison of reported median lethal doses (LD_50_, μg venom/g mouse) for mice of 3FTx- and PLA_2_-predominant *Micrurus* venoms. Route of venom administration: i.v., intravenous; i.p., intraperitoneal; s.c., sub-cutaneous; tw, this work.

3FTx-Rich Venom	LD_50_	Reference	PLA_2_-Rich Venom	LD_50_	References
*M. altirostris*	i.p. 0.26–0.65	[41]	*M. fulvius*	i.v. 0.32 ± 0.12	[43]
				i.p. 2.60–4.40	[53]
*M. corallinus*	i.p. 0.25–1.35	[41]	*M. tener*	s.c. 4.4	[45]
				i.v. 0.78 ± 0.14	[54,55]
*M. mipartitus*	i.p. 0.47	[52]	*M. nigrocinctus*	i.v. 0.3–0.5	[45]
				i.p. 0.4–1.2	
				s.c. 1.7–2.5	
*M. multifasciatus*	i.p. 1.35	[52]	*M. mosquitensis*	i.v. 0.20–0.61	[18]
*M. alleni*	i.v. 0.23–0.55	[18]	*M. dumerilii*	i.p. 0.8–1.9	[40]
	i.v. 0.74 ± 0.16				
*M. tschudii*	i.p. 0.44–0.81	[tw]			
*M. frontalis*	i.p. 0.20–1.45	[53]			
*M. pyrrhocryptus*	i.p. 1.10 ± 0.10	[51]			
*M. surinamensis*	i.p. 2.15–4.35	[53]			
*M. clarki*	i.v. 0.42–1.38	[46]			

## Supplementary Materials

The following are available online at https://www.mdpi.com/2072-6651/8/6/178/s1. Table S1: Summary of the MS/MS identification of the SDS-PAGE protein bands of *M. t. tschudii* venom.
